# Bioengineering of bacterial pathogens for noninvasive imaging and *in vivo* evaluation of therapeutics

**DOI:** 10.1038/s41598-018-30806-x

**Published:** 2018-08-22

**Authors:** Sathish Rajamani, Kyle Kuszpit, Jennifer M. Scarff, Linnea Lundh, Maisha Khan, Jennifer Brown, Robert Stafford, Lisa H. Cazares, Rekha G. Panchal, Thomas Bocan

**Affiliations:** Molecular & Translational Sciences Division, US Army Medical Research Institute of Infectious Diseases, 1425 Porter Street, Frederick, MD 21702 United States

## Abstract

Critical bacterial pathogens of public health and biodefense concerns were engineered to constitutively express *Escherichia coli* enzyme thymidine kinase (TK) that allows for noninvasive nuclear imaging via phosphorylation and entrapment of radiolabeled nucleoside analog 1-(2′deoxy-2′-fluoro-β-D-arabinofuranosyl)-5-iodouracil (FIAU). Expression of functional TK was established using a nucleoside analog Zidovudine that impeded the growth of *tk*-engineered bacteria. Significantly, no observable growth differences were detected for FIAU. High resolution mass spectrometry with *Pseudomonas aeruginosa* PAO1 and its *tk* variant (PAO1TK) confirmed FIAU phosphorylation and retention only in PAO1TK. *In vitro* gamma counting with wild-type PAO1, *Acinetobacter baumannii* and *Burkholderia pseudomallei* Bp82 and their *tk* derivatives with [^18^F]FIAU further confirmed that *tk* variants selectively incorporated the radiotracer, albeit with varying efficiencies. *In vitro* [^18^F]FIAU labeling coupled with *in vivo* Positron Emission Tomography/Computed Tomography (PET/CT) imaging of PAO1 and PAO1TK confirmed that only PAO1TK can be imaged in mice at sensitivities ≥10^7^ bacteria per infection site. This was further verified by administering [^18^F]FIAU to animals infected with PAO1 and PAO1TK. Utility of *tk*-engineered *P. aeruginosa* in noninvasive PET/CT imaging for bacterial therapeutic evaluation in animals was demonstrated employing antibiotic ciprofloxacin, underscoring the immediate use of PAO1TK and potentially other engineered pathogens for evaluating experimental therapeutics.

## Introduction

According to Centers for Disease Control and Prevention (CDC) report (2013), in the United States, over 2 million people annually contract serious bacterial infections that are untreatable by one or more of the antibiotics that were previously effective against these infections^1^. At least 23,000 people die per year as a direct result of these antibiotic-resistant infections, with many more mortalities due to complications caused by these infections. Inherent resistance of certain bacterial species to antibiotics and their ability to further develop resistance to previously effective antibiotic(s) have aggravated antimicrobial resistance (AMR) problems. The Infectious Disease Society of America has defined *Enterococcus faecium, Staphylococcus aureus, Klebsiella pneumoniae, Acinetobacter baumannii, Pseudomonas aeruginosa* and *Enterobacter* spp., abbreviated ESKAPE, as critical AMR pathogens. These pathogens are adept at ‘escaping’ the biocidal action of antibiotics and collectively represent new paradigms in pathogenesis, transmission and resistance^2^. Importantly carbapenem-resistant *P. aeruginosa* and *A. baumannii* are priority 1 “critical” agents in the World Health Organization (WHO) AMR priority listing^3^.

*P. aeruginosa* is a well-adapted, Gram-negative opportunistic pathogen that causes a wide range of acute and chronic infections in humans leading to high mortality rates. *P. aeruginosa* is recognized as a ‘superbug’ because it can actively develop resistance to multiple antibiotics. It is the second most common cause of all hospital-acquired bacterial infections and often the cause of wound infections. In addition, it is the most important cause of morbidity and mortality in cystic fibrosis patients (>80%). *A. baumannii*, a causative agent of wound infection, bacteremia, pneumonia, meningitis, and urinary tract infection, has become a serious problem in health care settings^4^. Multi-drug resistant (MDR) strains of *A. baumannii* have become increasingly prevalent, and the emergence of colistin-resistant strains presents a dire problem of very limited treatment options for eradication of infection^4^. Among these ESKAPE pathogens, *P. aeruginosa* and *A. baumannii* are the only pathogens that do not contain an endogenous thymidine kinase (*tk*) gene, making them ideal *tk* engineering candidates.

In addition, the *Burkholderia* genus contains a number of pathogens that cause important diseases, with some being a concern for our national security. For instance, *B. pseudomallei* is listed by the CDC as a potential agent of bioterrorism. *B. pseudomallei* (BSL-3, Class B agent) causes melioidosis and is notoriously resistant to a number of antibiotics which leads to limited treatment options^5^. Mortality rates in infected individuals are high owing to its intracellular lifestyle and sustained ability to resist antimicrobials, often confounded by infection remission even after extended antibiotic treatment. Many *Burkholderia* pathogens have no endogenous *tk* gene. The ability to engineer *Burkholderia* pathogens that express thymidine kinase enzyme (TK) will serve as useful tools for *in vivo* imaging to monitor the bacterial biodistribution and therapeutic potential of useful drugs in animal models.

Noninvasive preclinical and clinical *in vivo* imaging such as positron emission tomography (PET), single photon emission computed tomography (SPECT), magnetic resonance imaging (MRI), optical imaging, etc., are used to study disease pathology, biodistribution of pathogens, disease diagnostics and for evaluating therapeutic efficacy^6^. Bacterial pathogens expressing endogenous TK enzyme have been successfully imaged by PET/SPECT using radiolabeled [^124^I] FIAU/[^125^I] FIAU substrate via phosphorylation and entrapment in cells^7,8^. Importantly, *tk* is not conserved across all bacterial species, and its absence in some critical human pathogens limits their imaging potential. *Mycobacterium tuberculosis* modified to express bacterial TK was shown to be useful for SPECT/CT imaging using [^125^I]-FIAU^9^. Given the longer half-life of ^124^I and ^125^I labeled FIAU, images were collected for several hours or days post-infection and radiotracer injection. Prior bacterial imaging studies conducted with an initial bacterial load of 10^8^–10^9^ CFU/infection site, presumably had a substantially increased bacterial counts at the time of imaging, thus inflating the signal intensities obtained from infection sites. Engineering bacteria to express the bioreporter genes such as TK and using a shorter-lived isotope like [^18^F] might rapidly inform us of bacterial load and biodistribution in the host. Furthermore, *in vivo* imaging of pathogens to evaluate the efficacy of therapeutics offers useful drug evaluation models for studying critical pathogens, particularly with the rapid rise in AMR in bacteria.

Given the need for more noninvasive means of tracking bacterial infection, the objectives of the current study were set to: (i) demonstrate the *tk* bioengineering potential in three critical pathogens - *P. aeruginosa, A. baumannii* and *B. pseudomallei*, that lack the *tk* gene*;* (ii) utilize PET imaging to determine the sensitivity of detection at a given bacterial infection site within a reasonable time post-infection and (iii) establish the utility of bioengineered pathogens for developing *in vivo* therapeutic imaging model(s) for noninvasive evaluation of drug candidates in the host.

## Results

### Bacterial bioengineering and *in vitro* characterization of thymidine kinase expression

Genome-wide translated BLAST (BLASTX) analysis of select key pathogens with the *E. coli tk* gene as the query sequence identified the lack of conservation of this gene in number of critical pathogens, including *P. aeruginosa, A. baumannii and B. pseudomallei*. We assessed the possibility of engineering *tk* variants of three key pathogenic bacterial species lacking endogenous TK: *P. aeruginosa* PAO1 (PAO1), *Acinetobacter baumannii* ATCC 17978 (AB) and an avirulent derivative of a biothreat agent *Burkholderia pseudomallei* Bp82 (Bp82) (Δ*purM*, a purine auxotroph and a BSL-2 variant of the pathogen). In PAO1, the *E. coli tk* gene was fused to a constitutive native promoter *lasR* to achieve steady expression and accumulation of thymidine kinase (TK)^10^. Similarly in AB, the *E. coli tk* gene was fused with a constitutive native promoter of 16S ribosomal RNA with an engineered ribosomal binding site. For Bp82 expression, the *E. coli tk* was codon optimized and fused to native ribosomal protein S12 promoter to drive constitutive TK expression. PAO1 and AB contain one functional *att*Tn7 neutral site downstream of an essential gene, glutamine-fructose-6-phosphate aminotransferase (glmS in PAO1 and glmS2 in AB), for the mini-Tn7 cassette containing promoter-*tk* fusion and antibiotic cassette integration^11,12^. Bp82 has three functional *att*Tn7 sites downstream of three *glmS* genes designated *glmS1*, *glmS2* and *glmS3* for mini-Tn7 cassette integration, with no accountable differences between these sites for gene engineering^13^. Individual colonies selected on respective selection plates were verified using target-specific PCR to confirm the proper integration of the *tk* gene cassette within the *att*Tn7 site(s). For Bp82, a representative strain with *tk* integrated at *glmS3 att*Tn7 site was used for further characterization.

**Figure 1:**
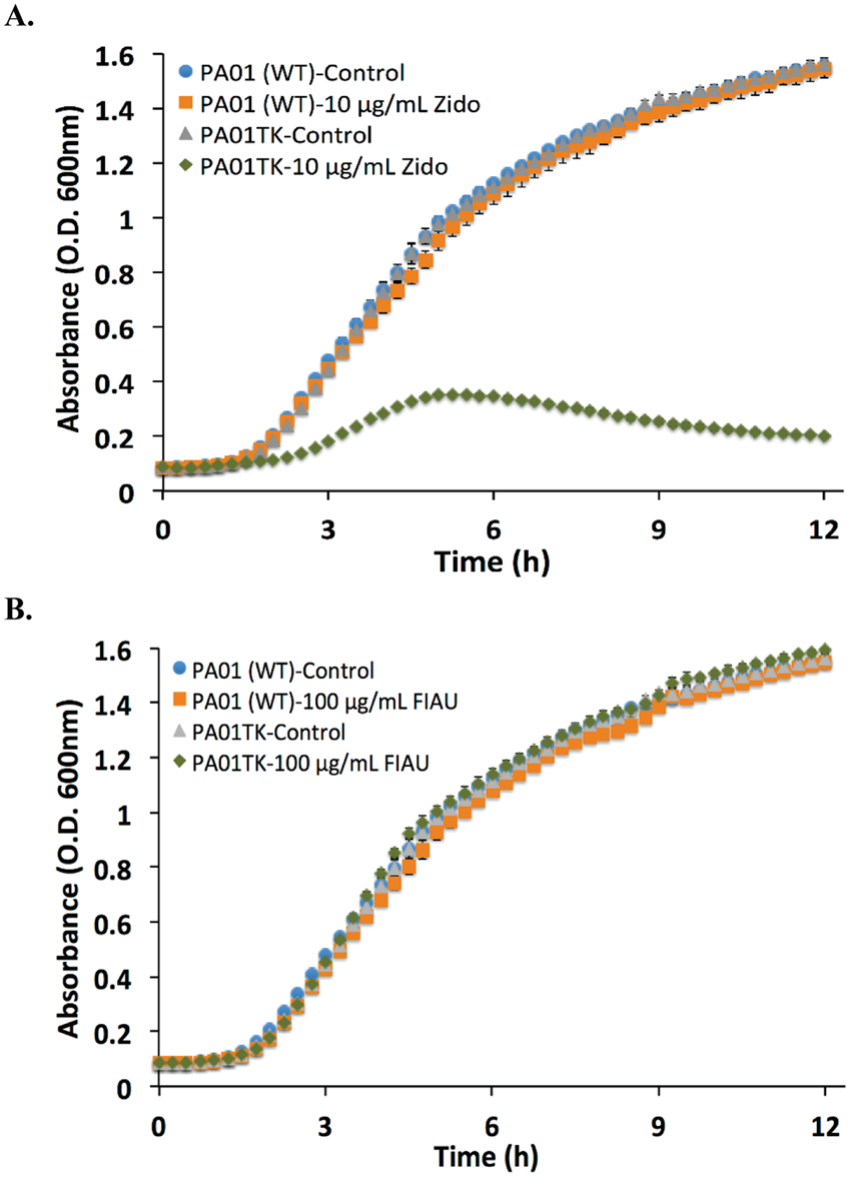
Growth curve of *Pseudomonas aeruginosa* (PAO1) and PAO1 *tk-*derivative (PAO1TK) in the presence and absence of nucleoside analogs. Absorbance (OD_600_) of PAO1 or PAO1TK grown in the presence of (**A**) Zidovudine (Zido, 10 µg/mL) or (**B**) 1-(2′deoxy-2′-fluoro-β-D-arabinofuranosyl)-5-iodouracil (FIAU, 100 µg/mL).

Previously, *E. coli* expressing endogenous TK, but not a *tk*-deficient strain, showed growth inhibition with the addition of nucleoside analogs Zidovudine and 1-(2′deoxy-2′-fluoro-β-D-arabinofuranosyl)-5-iodouracil (FIAU) which is due to TK phosphorylation and entrapment of these substrates^7^. The exact mechanism behind the entrapped phosphorylated nucleoside’s role in causing bacterial growth inhibition remains to be elucidated. Based on this knowledge, we subjected our *tk*-engineered bacteria and wild-type counterparts to Zidovudine or FIAU and monitored their effects on *in vitro* growth. As shown in Fig. 1A, PAO1TK, but not wild-type PAO1, had a growth inhibition when incubated with Zidovudine (10 µg/mL), confirming the production of functional TK in PAO1TK cells. However, no growth inhibition compared to wild-type cells was observed in PAO1TK in the presence of 100 µg/mL FIAU (Fig. 1B). Similar observations were made with ABTK and Bp82TK grown with Zidovudine and FIAU (SI Figs. 1 and 2).

### Mass Spectrometric analysis for detection of phosphorylated FIAU

**Figure 2:**
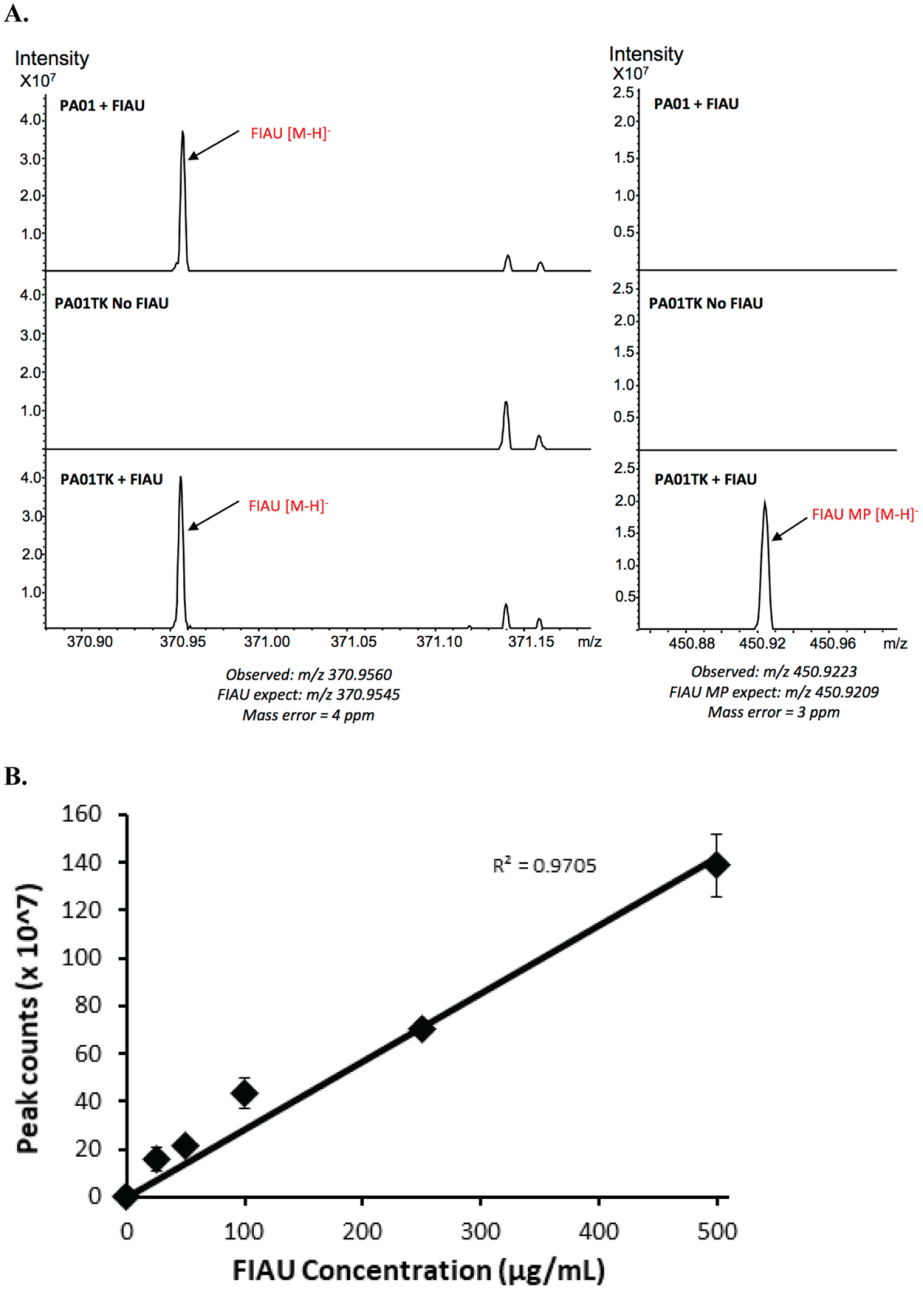
Mass spectrometric analysis to detect and quantify phosphorylated FIAU in *Pseudomonas aeruginosa* (PAO1) and PAO1 *tk-*derivative (PAO1TK). (**A**) PAO1 and PAO1TK were incubated with FIAU and MALDI-FT-ICR-Mass Spectrometry was performed on unwashed (left panel) or washed (right panel) cells. The regions in the spectra corresponding to peaks where FIAU (m/z 370.95) or monophosphorylated FIAU (FIAU-MP, m/z 450.92) were detected are labeled. (**B**) The amount of FIAU-MP that accumulated in washed PAO1TK cells grown with different concentrations of FIAU (25–500 µg/mL).

To address the fate of FIAU in *tk*-engineered bacteria, a Fourier transform ion cyclotron resonance mass spectrometry (FT-ICR MS) method for detecting FIAU or phosphorylated FIAU was developed. FIAU cold labeling studies with PAO1 and PAO1TK were conducted and the samples analyzed using FT-ICR MS. All *P. aeruginosa* unwashed cells incubated with FIAU exhibited a peak in the spectrum that corresponded with the expected mass for FIAU within 4 ppm accuracy (m/z 370.9560). PAO1TK, but not PAO1, also had a peak that corresponded with monophosphorylated FIAU (FIAU-MP) at m/z 450.9223 with 3 ppm accuracy (Fig. 2A, left panel). Washing the PAO1 and PAO1TK cells removed unphosphorylated FIAU, as demonstrated by the absence of a corresponding mass ion for FIAU, which indicates that FIAU is not retained within cells. However, FT-ICR-MS analysis detected a mass ion peak for FIAU-MP in washed PAO1TK cells, which confirms the cellular retention of the phosphorylated TK substrate (Fig. 2A, right panel). Importantly, for PAO1TK, we also observed a dose-dependent increase in FIAU-MP with the addition of increasing concentrations of FIAU (25 µg/mL–500 µg/mL) (Fig. 2B), indicating that FIAU likely diffuses freely into or is actively taken up by PAO1TK cells.

### Labeling of *tk*-engineered bacteria with [^18^F]FIAU

**Figure 3:**
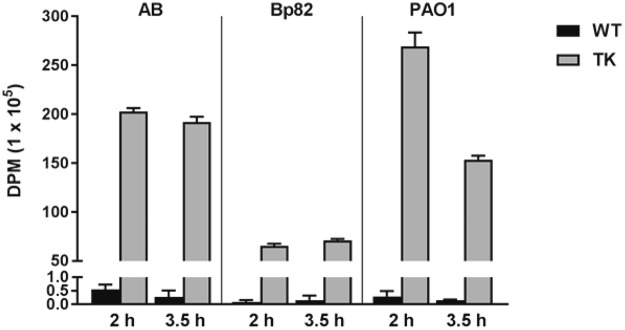
*In vitro* [^18^F]-FIAU labeling of wild-type and *tk*-engineered *A. baumannii* (AB)*, B. pseudomallei* (Bp82) and *Pseudomonas aeruginosa* (PAO1). Bacteria (10^9^ CFU/mL in PBS) were incubated with [^18^F]-FIAU (150 µCi/mL) for 2 h or 3.5 h and the washed cells were analyzed by a Gamma counter to determine the amount of radioactivity disintegrations per minute (DPM) present in the cells. One-way ANOVA analysis between wild-type (WT) and cognate *tk-*derivatives (TK) established statistically significant difference (P < 0.0001) in radioactivity accumulation at each time point.

As the specific activity of radiolabelled [^18^F]FIAU used for imaging studies is 1–5 Ci/µmol and the resultant FIAU concentration is in the nanoMolar range, we wanted to further validate the FIAU uptake and phosphorylation at this low concentration. To determine these efficiencies, the wild-type and the *tk*-engineered bacterial strain counterparts resuspended in phosphate buffered saline (PBS) were incubated *in vitro* with a single dose of [^18^F]FIAU and samples collected at 2 h and 3.5 h. The radioactivity associated with each sample was recorded using a gamma counter. As shown in Fig. 3, our data clearly shows that the TK derivative strains had several hundred-fold increases in radioactivity compared to their respective parent strains at both time points. At 2 h and 3.5 h, the DPM accumulation increased 369-fold and 712-fold, respectively, in ABTK compared to AB. A similar increase in radioactivity was observed at 2 h and 3.5 h for Bp82TK, 818-fold and 474-fold, as well as PAO1TK, 929-fold and 1023-fold, respectively, compared to their wild-type controls (Fig. 3). While the DPM accumulation remained comparable between 2 h and 3.5 h in ABTK and Bp82TK, there was about 43% decrease in the accumulation observed for PAO1TK between 2 h (269 × 10^5^ DPM) and 3.5 h (153 × 10^5^ DPM). Importantly, earlier characterization of PAO1TK using growth analysis and mass spectrometric FIAU incubation studies in nutrient replete media showed no loss in growth cell density or any indication of FIAU dephosphorylation at high doses of FIAU even at longer incubation periods (6–12 h, see Figs 1 and 2). Given that a number of researchers have successfully employed radiolabeled-FIAU for bacterial imaging applications with diverse bacterial strains and with studies spanning hours to days without loss of entrapped phosphorylated-FIAU^7–9^, we believe that the *in vitro* PAO1TK radiolabeling loss is directly linked to the design of this experiment. The resuspension of bacterial cells in nutrient free PBS to limit bacterial replication potentially impacted the [^18^F]FIAU labeling outcome of PAO1/PAO1TK more so than AB/ABTK and Bp82/Bp82TK. It is our speculation that over time, nutrition deplete conditions could have resulted in the loss of phosphorylated [^18^F]FIAU labeled PAO1TK cells possibly via cell death and lysis. Significantly, as detailed below, we did not notice any loss of [^18^F]FIAU radioactivity signal in our animal studies with PAO1TK, even when images were collected over 5 h post-radiotracer injection (SI Fig. 3), further alleviating our concern.

### PET/CT imaging of mice infected with [^18^F]FIAU pre-labeled bacteria

**Figure 4:**
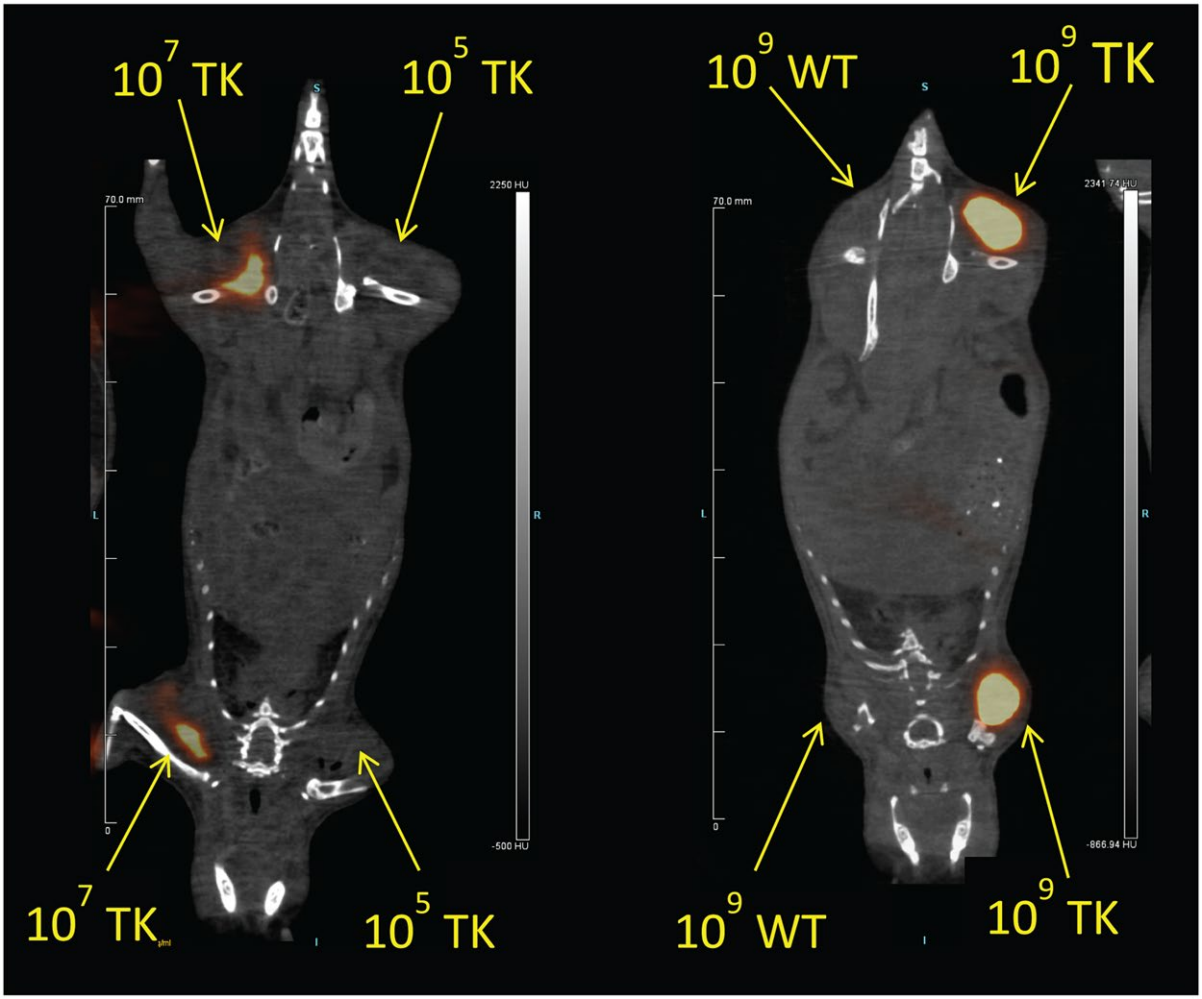
*In vivo* PET/CT imaging of [^18^F]FIAU pre-labeled *Pseudomonas aeruginosa* (PAO1) and PAO1 *tk-*derivative (PAO1TK). CD-1 mice (n = 3 mice/group) were injected i.m. with 10^5^, 10^7^ or 10^9^ CFU PAO1 (WT) or PAO1TK (TK) that were pre-incubated with [^18^F]FIAU for 2 h and subjected to PET/CT imaging immediately. Detectable bacterial signal intensity was observed in both the forelimb and hindlimb that were injected with either 10^7^ and 10^9^ CFU but not 10^5^ CFU. Data shown here are representative images from this experiment.

To determine the *in vivo* limits of detection of [^18^F]FIAU labeled bacteria, CD-1 mice were injected in the thigh with 10^5^, 10^7^ or 10^9^ CFU of pre-labeled wild-type or *tk*-engineered PAO1 and PET imaging was performed immediately (Fig. 4). While there was no discernable PET signal that colocalized to the site of injection with any dose of PAO1 and 10^5^ CFU PAO1TK, there was a distinct PET signal in the thighs injected with 10^7^ and 10^9^ CFU PAO1TK. These studies suggest that the detection limit for [^18^F]FIAU and PAO1TK PET imaging is ≥10^7^ CFU per site of infection.

### PET imaging of mice infected with wild-type and *tk*-engineered PAO1

**Figure 5:**
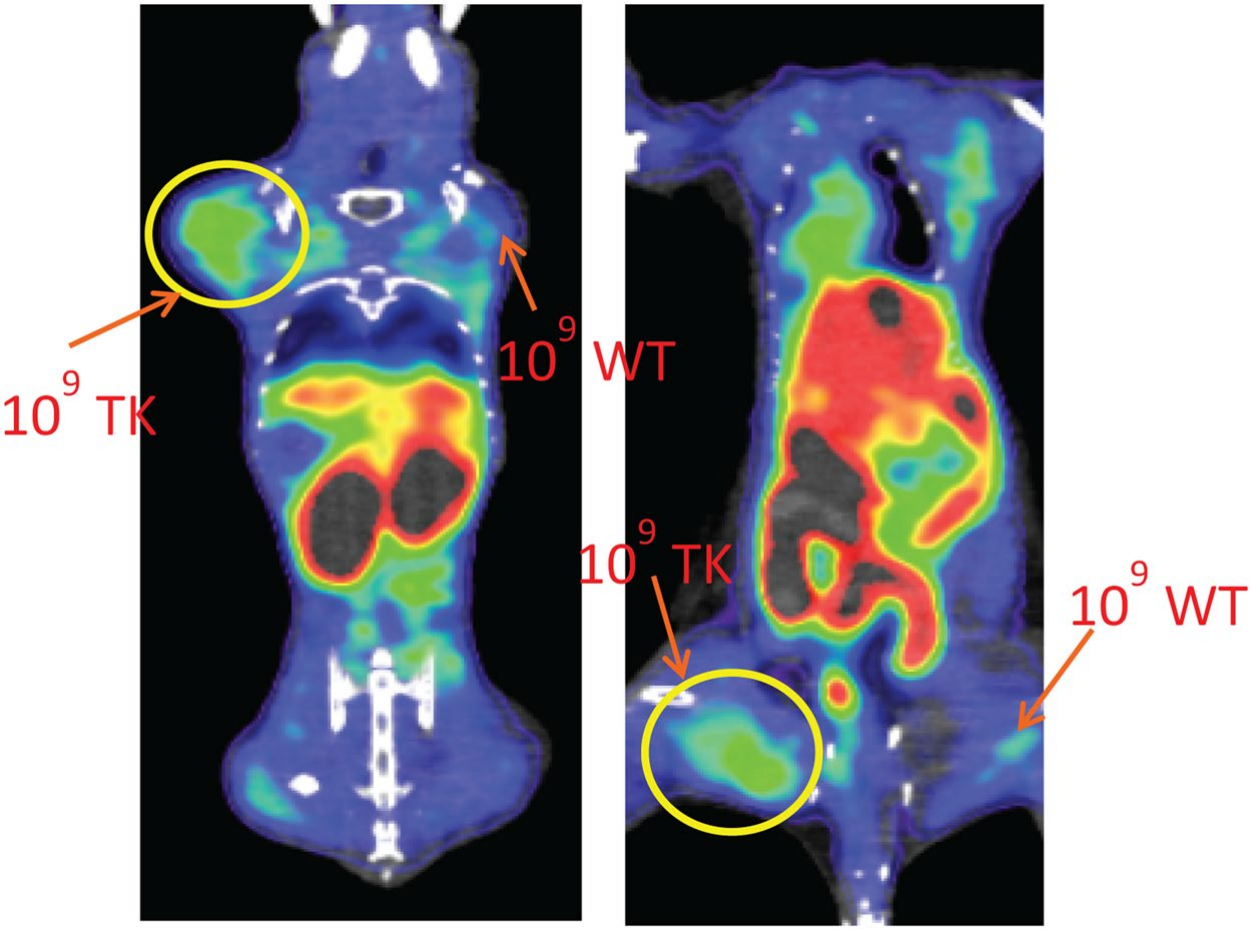
*In vivo* PET images of mice infected with *Pseudomonas aeruginosa* (PAO1) and PAO1 *tk-*derivative (PAO1TK). Two BALB/c mice were i.m. infected with 10^9^ CFU PAO1 (10^9^ WT) or 10^9^ CFU PAO1TK (10^9^ TK) 2 h prior to i.v. injection with [^18^F]FIAU. WT (right upper and lower quadrants) infected mice and TK (left upper and lower quadrants) infected mice were subjected to PET/CT imaging 2 h post radiotracer injection. To obtain best views of the bacterial infection sites, the image planes showing upper and lower limbs of the animal were threshold adjusted to obtain the desired plane. Both the animals showed similar observations corresponding to injected bacteria. Data shown here are representative images from this experiment. Independent repeats of this experiment resulted in similar observation.

The utility of our bioengineered pathogens for *in vivo* PET imaging was further evaluated with a similar *P. aeruginosa* thigh infection model. BALB/c mice were injected via the intramuscular (i.m.) route with PAO1 or PAO1TK (10^5^, 10^7^ or 10^9^ CFU) in the forelimbs and hindlimbs. After 2 h, [^18^F]FIAU was injected via the tail vein, and dynamic PET imaging was performed over a period of 5 h. PAO1TK, but not PAO1, at a dose of 10^9^ CFU could be clearly detected at the site of infection (Fig. 5). Lower bacterial doses of PAO1TK were also tested and there was no detectable bacterial signal observed with 10^5^ CFU and only a weak signal was observed with 10^7^ CFU (SI Fig. 4). As evident from these images, there is a great deal of background signal observed in animals injected with [^18^F]FIAU (Fig. 5), compared to mice injected with pre-labeled bacteria (Fig. 4). This is primarily attributed to biodistribution and metabolism of the radiotracer in the host, particularly in the gastrointestinal tract of the animal. Dynamic scans with 10^9^ CFU PAO1TK showed that the background radiotracer biodistribution is visible even at ~4 h post-injection of radiotracer (SI Fig. 3). Importantly, an unambiguous signal from injection sites with 10^9^ CFU PAO1TK was noticeable as early as 60 min after [^18^F]FIAU administration (SI Fig. 3). Similar observations were also made at 6 h post-administration with the longer half-life radiotracer, [^125^I]FIAU^7^. Our studies indicate that useful measurements of infected sites in the mouse can be recorded between 2–4 h post [^18^F]FIAU injection despite high background.

### Noninvasive therapeutics evaluation in mice infected with *P. aeuroginosa*

**Figure 6:**
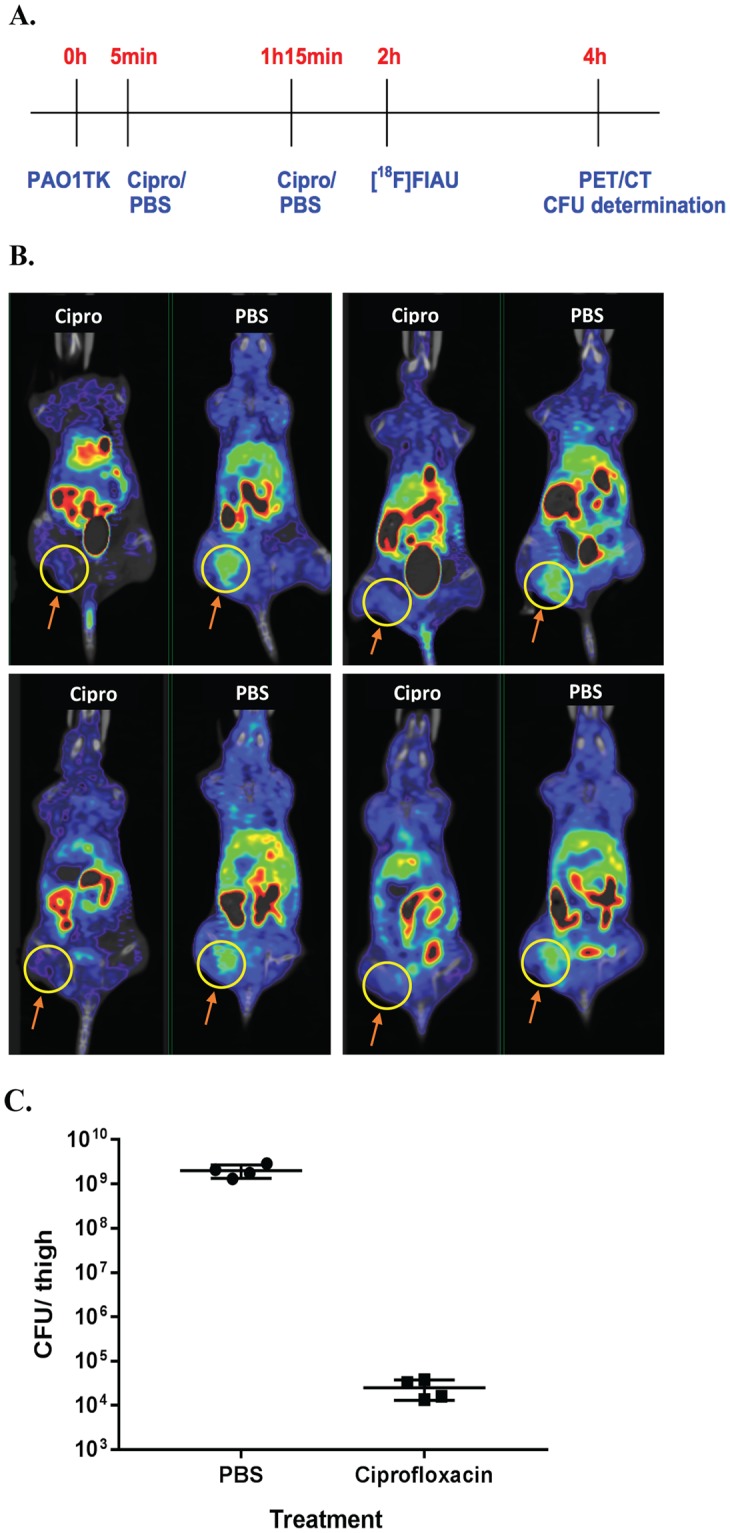
*In vivo* PET bacterial imaging and therapeutic efficacy evaluation. (**A**) Timeline of experiment (not to scale): Mice (n = 8) were infected with 10^9^ CFU PAO1TK, four each were treated with Ciprofloxacin (Cipro, 40 mg/kg) or PBS i.p., at 5 min and 75 min post-infection, injected i.v. with ~250 µCi [^18^F]FIAU at 2 h post-infection, and imaged at 4 h post-infection (2 h post-radiotracer administration). **(B**) PET/CT scans of all PBS and Cipro-treated mice. The thigh infected with PAO1TK is circled in yellow. (**C**) The bacterial load of PAO1TK in the thighs of mice treated with PBS or Ciprofloxacin. Unpaired t-test between the treatment groups showed significant difference in bacterial CFU (P = 0.0010). These experiments were repeated independently resulting in similar observations.

Since PET imaging proved to be a useful tool for monitoring bacterial load, we sought to design a therapeutic evaluation model in mice following PAO1TK infection. For our proof-of-concept study, we selected ciprofloxacin to evaluate bacterial susceptibility and loss of radiotracer bacterial labeling in mouse thigh infection model. Ciprofloxacin or PBS was administered to mice post-infection with 10^9^ CFU PAO1TK, followed by [^18^F]FIAU injection to determine if PET images could correlate with differences in bacterial load (Fig. 6A). Mice (n = 4) that received PBS had a strong PET signal corresponding to the site of injection of PAO1TK at 4 h post-infection. However, mice that received ciprofloxacin showed a substantially lowered PET signal at the site of bacterial infection (Fig. 6B). We also observed a 4–5 log reduction in bacterial load in the thighs from ciprofloxacin-treated mice compared to the thighs from the PBS-treated control mice (Fig. 6C).

## Discussion

Increased incidence of AMR, including emergence of extreme drug resistant (XDR) or pandrug resistant (PDR) microbes deemed “superbugs,” is a serious concern. Widespread use of antibiotics has augmented the prevalence of these pathogens in diverse environmental niches such as hospitals, meat and dairy farms, soil, water, etc.^14^, increasing the chance of MDR, XDR or PDR infections. In 2013, the CDC reported that annual cost due to infections caused by AMR pathogens is over $20 billion, with patients spending more than 8 million additional days in the hospital for treatment^1^. This accounts for substantial economic losses due to lost work hours and productivity. Further, the existence of natural resistance in key bacterial biothreat agents, such as *B. pseudomallei*, has contributed to a high incidence of remission and 10–40% mortality rates in melioidosis patients^5^. Newer strategies are being proposed and investigated for bypassing bacterial drug resistance barriers^15^, but the rate of their development is excruciatingly slow. If AMR in pathogens is not addressed immediately we will be in imminent danger of widespread untreatable infections that could have devastating public health, national defense, and economic consequences. A great deal of work is focused on understanding AMR mechanisms, developing novel therapies and animal model development for therapeutic evaluation. Present state-of-the-art therapeutic efficacy testing relies heavily on euthanizing the animal prior to bacterial isolation from the infected sites for bacterial enumeration. This is quite labor intensive and would typically require the use of several animal cohorts at multiple time points for studying drug efficacy.

Significantly, the lack of native *tk* in critical pathogens limits the possibility of infection model development using radiolabelled FIAU and PET imaging. We successfully demonstrate that stable chromosomal integration of promoter-*tk* fusions at a neutral site (*att*Tn7) can be developed for three different critical pathogens and show functional expression of TK (*in vitro* labeling, Fig. 3). The pUC18TminiTn7 plasmid derivatives containing *tk* engineering cassettes for neutral site *att*Tn7 chromosomal integration can be readily adapted for modifying other bacterial strains, including critical MDR, XDR, or PDR strains. In instances where there is not a useful antibiotic selection marker for pathogens under investigation, other reporter genes in place of an antibiotic marker (such as *gfp*, *rfp*, luciferase gene, *etc*.) can be adapted for initial identification of the engineered sub-population of bacteria.

We observed growth inhibition of our *tk*-engineered pathogens when incubated with Zidovudine. Incubation of PAO1TK with FIAU had no effect on growth, although we confirmed by FT-ICR-MS analysis that cells retained FIAU-P. This could be primarily attributed to the differences in the nucleoside analog substrates. It is possible that the phosphorylated Zidovudine might be incorporated into bacterial DNA during synthesis which causes alterations and growth inhibition, whereas FIAU-P may not readily be incorporated into DNA and causes no discernable growth inhibition. Significantly, these assays confirmed that our pathogens expressed functional TK and that our engineering method could be employed to assess other Gram-negative bacteria.

*In vitro* labeling and gamma counting studies indicated that all the pathogens bioengineered to express TK trapped the [^18^F]FIAU. Significantly, there was an overall decrease in PAO1TK labelling from 2 h to 3.5 h (Fig. 3). We speculate that at 3.5 h, PAO1TK-[^18^F]FIAU-P labeled cells may have lysed during sample processing or PAO1 resorts to undetermined mechanism (efflux, dephosphorylation of radiotracer resulting in exit, etc.) under the *in vitro* labeling conditions to reduce the concentration of [^18^F]FIAU-P trapped within the cells. Importantly, as shown in our studies this observation had no significant bearing on the outcome of PAO1TK bacterial *in vivo* imaging.

We established that the detection limit of PAO1TK in PET imaging is about 10^7^ CFU per infection site. We also determined that there is no demonstrable signal contribution from the uninfected sites or from wild-type bacteria. Our limit of detection is in agreement with the previously published SPECT detection limit of *tk*-engineered *Mycobacterium tuberculosis*^9^. The lowest bacterial load that has been detected using FIAU substrate was 2 × 10^6^ CFU/gram tissue in mice infected with *Staphylococcus aureus* strain 25933, which has endogenous TK expression^7^. In order to improve bacterial imaging sensitivities, ^18^F radiolabeled derivatives of maltohexose and sorbitol substrates have been employed and were shown to have moderate success in improving the *E. coli* detection limit to about 10^5^ and 1.6 × 10^6^ CFU, respectively^16,17^. In these instances, as noted for sorbitol^17^, the bacterial strains presumably utilized these carbohydrates for metabolism and have a mechanism to take up and entrap (via phosphorylation) the radiolabelled substrate. The use of radiolabeled metabolic sources has the same limitations as TK-based imaging, since some pathogens, such as *P. aerugonisa*, lack the ability to take up sorbitol. Although optical imaging with engineered bacterial bioreporters, e.g., green fluorescent protein, luciferase-luciferin, have shown higher sensitivities for bacterial detection (<10^4^ CFU), the images acquired are often 2-dimensional and the imaging is limited in its depth of tissue penetration. Additionally, optical imaging, unlike PET/CT requires nude or non-pigmented mice. This allows for PET/CT imaging, but not optical imaging, to be used in preclinical evaluations that require the use of pigmented larger mammals, including non-human primates^6^.

We observed non-specific biodistribution and a high accumulation of [^18^F]FIAU in the host gastrointestinal tract consistently during our PET/CT imaging studies after radiotracer application. Similar observations were made by groups using [^125^I]FIAU for bacterial SPECT imaging^7,9^. Interestingly, studies employing longer-lived radiolabelled FIAU (such as ^125^I with half-life of 59.5 days compared to ^18^F half-life of 2 h) demonstrated that the non-specific signal within the gastrointestinal tract and kidneys of the host diminished with time, which improved bacterial signal detection at the infection site^7^. Though longer-lived tracers offer advantages for extended PET/ SPECT imaging, previous studies that monitored mice over 12 h to 120 h post-infection with 10^8^ or 10^9^ CFU had no correlating bacterial numbers at the time of imaging. The bacterial counts in these studies were possibly much higher than what was initially injected in the animal. This is further underscored from our observation that mice infected with 10^9^ CFU of PAO1TK showed about 2-fold increase in bacterial count within 4 h post infection (10^9^ CFU to 2 × 10^9^ CFU, see Fig. 6C), emphasizing the need for rapid bacterial imaging and detection. In our antibiotic assessment study, the loss of detectable PET signal in antibiotic treated animals correlated well with the reduction in PAO1TK CFU counts below our established detection limit of ≥10^7^ CFU.

In summary, bioreporters of bacterial pathogens offer useful tools for studying infection pathology, including spread of infection, identifying bacterial reservoirs in tissues and monitoring the effect of antibiotics on the bacterial population. We engineered bacteria to express a non-native TK for use as a bioreporter. We utilized bacterial imaging models for detecting bacteria, and evaluating therapeutics in live noninvasive mouse models. This further exemplifies the possibility of using *in vivo* imaging techniques to rapidly test the efficacy of next-generation antimicrobials or novel antimicrobial combinations in animals.

## Methods

### Bacterial strains and growth conditions

The bacterial strains used in this study, *Escherichia coli* (DH5α, ATCC 25922), *Pseudomonas aeruginosa* PAO1 (ATCC 15692), *Acinetobacter baumannii* (ATCC 17978) and avirulent /non-BSAT strain of *Burkholderia pseudomallei* Bp82 (Δ*purM*) (generously made available by Dr. Herbert P. Schweizer, University of Florida), were grown in LB broth and on LB agar (EMD Chemicals, Gibbstown, NJ) plates. *E. coli* BW29427 (generously provided by Dr. Diane Newmann, Caltech, Pasadena, USA) was grown in LB media supplemented with 360 µM of 2,6 diaminopimelic acid (DAP). Unless otherwise stated, antibiotics were supplemented in the media at the following final concentrations for bacterial selection: 100 µg/mL ampicillin (Ap), 50 µg/mL Kanamycin (Km), 10 µg/mL Gentamicin (Gm) for *E. coli*; 30 µg/mL Gm for *P. aeruginosa*; 1,000 µg/mL Km for *B. pseudomallei* Bp82 and 50 µg/mL Gm for *A. baumannii*.

### DNA manipulation and bacterial engineering with thymidine kinase (*tk*)

The gene for thymidine kinase (*tk*) was inserted into the chromosome of PAO1, *A. baumannii* (AB) and Bp82 to yield strains PAO1TK, ABTK and Bp82, respectively, as described below. DNA sequences of the promoter-*tk* gene fusions and DNA primers used for *tk* gene engineering are listed in the supplementary information (S. Info. 1–3). Briefly, the *tk* construct for *P. aeruginosa* was created by cloning *P. aeruginosa* promoter *lasR* (428 bp region upstream of *lasR* ORF, GenBank: M59425.1) into pBBR1MCS4^18^ using KpnI and ClaI sites and sequentially fused to *E. coli tk* gene (*tdk -*GenBank: DQ384607.1). *E. coli tk* gene was PCR amplified from *E. coli* ATCC 25922 genome and cloned downstream of P_lasR_ using ClaI and XbaI sites. The full-length fusion of P_lasR_-*tk* was subcloned into mini-Tn7 vector pUC18T-mini-Tn7T-Gm (GenBank Accession #AY599232)^11^ using BamHI/HindIII sites creating pUC18T-mini-Tn7T-Gm- P_lasR_-*tk*. For AB, a native promoter fusion of 16S rRNA gene (327 bp, GenBank: CP000521.1) with a ribosomal binding site and *E. coli tk* was obtained by gene synthesis (Genewiz, NJ, USA). The resulting P_16SrDNA_-*tk* fusion in pUC57 vector was used to excise P_16SrDNA_-*tk* fusion and subcloned into pUC18T-mini-Tn7T-Gm using BamHI/HindIII sites, creating pUC18T-min-Tn7T-Gm-P_16SrDNA_-*tk*. Similarly, *B. pseudomallei* native S12 gene promoter (205 bp, GenBank: CP004379.1) and *B. pseudomallei* codon-optimized *tk* gene fusion was synthesized (Genewiz). The resultant P_S12_-*tk* clone in pUC57 was used as a PCR template to amplify the P_S12_-*tk* and clone it into mini-Tn7 vector pUC18T-mini-Tn7T-Km::FRT^13^ using KpnI/HindIII sites, creating pUC18T-mini-Tn7T-Km::FRT-P_S12_-*tk*. Stable chromosomal integrated *tk* lines of PAO1 was generated by coelectroporation of pUC18T-mini-Tn7T-Gm-P_lasR_-*tk* and transposase encoding helper plasmid pTNS2^13^ into PAO1 competent cells and selection on LB-Gm agar plates. Single colony isolates of Gm resistant PAO1TK putative transformants were verified to contain PAO1 chromosomal *att*Tn7 neutral site integration using primers described elsewhere^11^. AB and Bp82 were chromosomally engineered with the *tk* gene using a triparental mating protocol. Briefly, a mixture of 1:1:1 ratio of *E. coli* BW29427 cells carrying respective mini-Tn7 plasmid, *E. coli* BW29427 carrying and recipient AB or Bp82 cells were incubated on LB-DAP plates for conjugation. After 24 h, the cell mixtures were collected and suspended in fresh LB broth and plated on LB-Gm and LB-Km agar plates for selection of AB and Bp82 respectively. Single colony isolates were obtained and putative ABTK strains were verified by PCR for chromosomal *att*Tn7 neutral site integration using primers Tn7R (New): 5′CCACGCCCCTCTTTAATACGACGG and AB_GlmS1 (new-2): 5′GCGCGTGGCGGTGAGTTATTC, that were modified from the ones described before^12^. As detailed in Choi *et al*.^13^ putative Bp82TK colonies, were PCR verified for one of the 3 *glmS* site (*glmS1*, *glmS2* and *glmS3*) with these modified primer sets: BpglmS1(New) 5′GCGAGGAGTGGGCGTCGATCAAC; BpglmS2(New) 5′CCGACACGACGCAAGAGCGGAATC; BpglmS3(old) 5′CGGACAGGTTCGCGCCATGC with PTn7L(New) 5ATTAGCTTACGACGCTACACCCAGTTCCC.

### *In vitro* growth characterization of *tk* engineered bacteria

Saturated cultures of wild-type and *tk*-engineered bacteria were diluted 1:100 in fresh media and used for evaluating FIAU and Zidovudine in bacterial growth assay. In a Bioscreen Honeycomb well plate (Fisher Scientific, USA), 200 µl of cultures were aliquoted into wells with desired concentrations of FIAU and Zidovudine. For controls, cultures without any compound treatment and bacteria free media were included in each assay plate. The plates were incubated at 37 °C with continuous shaking in a Bioscreen C MBR (Oy Growth Curves Ltd, Finland) and the OD_600_ was collected at 15 min intervals. The experiments were repeated two or more independent times to verify results.

### Fourier transform ion cyclotron resonance mass spectrometry (FT-ICR MS) analysis of bacteria cultured with FIAU

Saturated bacterial cultures (16 h) were diluted 1:100 in fresh LB media and the desired concentration of FIAU (0–500 µg/mL) was added to the culture. Cultures were incubated at 37 °C, 225 rpm, for 6 h. Cells were harvested from a 5 mL volume of culture by centrifugation at 1,600 × *g* and the cell pellet was washed with 10 mL of 1X phosphate buffered saline (PBS). The bacterial pellet was resuspended in a 1X final concentration of phosphatase inhibitor (Halt Phosphatase inhibitor cocktail, ThermoFisher Scientific). To the bacterial slurry, 800 µL of MS grade methanol was added and vortexed for 1 min. The cell pellet extract was centrifuged (16,000 × *g*) for 10 min at room temp and the supernatant was collected. The methanol extract was dried in a SpeedVac (Labconco corp.) and the sample was resuspended in 20 µL ammonium formate and diluted 1:5 in MS matrix 9-aminoacridine (9AA), and 1 µL of this was used for spotted on matrix-assisted laser desorption /ionization (MALDI) target and read via negative ion mode in a 7T-SolariX XR instrument equipped with a Smartbeam II laser (Bruker Daltonics, Billerica, MA) at a repetition rate of 1 kHz, over a mass range of 150–1000 Da. Calibration was performed before each run using sodium trifluoroacetate clusters, and on-line calibration using the 9AA matrix peak was performed during each run. A total of 300 laser shots were summed over 32 scans for each sample and accurate masses for FIAU and phosphorylated FIAU (FIAU-P) were determined using internal calibration using 9AA matrix ions, endogenous adenosine diphosphate (ADP) and adenosine triphosphate (ATP) ions.

### PET radiotracer, PET/CT imaging, data acquisition, image reconstruction and data analysis

[^18^F]FIAU was obtained from the Imaging Probe Development Center, NIH, Rockville, MD and was produced by employing slight modifications to procedures already reported^19^, and using a commercially available GE TRACERLab FX-N Pro synthesizer. Ready-to-inject, >99% radiochemically pure [^18^F]FIAU (formulated in physiological saline containing ~10% ethanol) was obtained with 30–40% (n = 12) non-decay-corrected yields and the specific activity at the end of the 70 min radiosynthesis ranged from 48 to 200 GBq/μmol.

PET/CT scanning was performed using an Inveon preclinical PET/CT system (Siemens Medical Solutions, Knoxville, TN) with a spatial resolution of ~1.5 mm full width at half maximum at the center of the field of view. Imaging experiments were conducted as detailed in a previous report^19^ with some modifications. Mice were anesthetized with isoflurane (4% induction, 2% maintenance) delivered in oxygen. Once anesthetized, animals were administered ~250 µCi of [^18^F]FIAU in ~150 µL volume via intravenous (i.v.) injection in the lateral tail vein and were allowed to wake in their home cages for a 120 min radiotracer uptake phase. For dynamic scans after radiotracer injections, the animals were allowed to wake in their home cages for 60 min radiotracer uptake phase and scanned every 60–75 min up to 5 h post-[^18^F]FIAU administration. Just prior to scanning, mice were anesthetized with isoflurane and PET imaging was performed.

All image reconstructions were performed using Siemens’ Inveon Acquisition Workplace v2.0 software package (Siemens Medical Solutions, Knoxville, TN). Hounsfield Unit (HU) calibrated CT data were reconstructed using a Feldkamp reconstruction algorithm with a Shepp-Logan reconstruction filter, slight noise reduction, and beam hardening correction applied. Decay-corrected PET images were reconstructed using an iterative OSEM3D/MAP algorithm with scatter, dead time, and CT-based attenuation correction. The reconstruction parameters were 18 subsets, 3 iterations in a 128 × 128 matrix with a target resolution of 1.2 mm. PET images were co-registered to corresponding CT data using VivoQuant v2.5 image processing software (inviCRO, LLC, Boston, MA). PET imaging data were reported in terms of percent injected dose per gram of tissue (%ID/g), calculated as a ratio of tissue radioactivity concentration (Bq/g) at time of scan to total injected activity (Bq) at time of scan.

### *In vitro* labeling of bacteria with [^18^F]FIAU

Saturated bacterial broth cultures of PAO1, PAO1TK, AB and ABTK were diluted 1:50 in fresh LB media, while Bp82 and Bp82TK were diluted 1:20 and grown for a period of 2–3 h in an incubator shaker at 23 °C and 250 rpm. Cell density was determined at OD_600_ and cell number enumerated. The culture was harvested by centrifugation at 1,610 × *g* for 20 min at 23 °C and the cell pellet resuspended in PBS to a density of 1 × 10^9^ CFU/mL. Each cell suspension was incubated with [^18^F]FIAU at concentration of 150 µCi/mL culture at 35 °C with shaking at 175 rpm. Samples were removed at 2 h and 3.5 h, placed in 1.5 mL microcentrifuge tubes and cells were harvested by centrifugation at 20,500 × *g* for 3 min. The cell pellet was washed twice with PBS, resuspended in PBS, and placed in a Gamma counter (Perkin Elmer, USA). The cell pellets were counted on the gamma counter until the count per min (CPM) were within the linear range of the detectors, i.e., approximately, 1.7 × 10^6^ CPM. The CPM were decay corrected to the time of addition of [^18^F]FIAU to the bacteria and the final data was expressed in disintegrations per min (DPM) which was calculated by dividing the CPM by the counting efficiency factor for ^18^F, namely, 0.61162, which was determined during the machine calibration process.

### PET/CT imaging of mice infected with *in vitro*-labeled bacteria

All mouse research were conducted under an IACUC-approved protocol in compliance with the Animal Welfare Act, PHS Policy, and other Federal statutes and regulations relating to animals and experiments involving animals. The facility where this research was conducted is accredited by the Association for Assessment and Accreditation of Laboratory Animal Care, International and adheres to principles stated in the Guide for the Care and Use of Laboratory Animals, National Research Council, 2011.

PAO1 and PAO1TK (2 × 10^9^ CFU/mL) were incubated with [^18^F]FIAU at a concentration of 150 µCi/mL and prepared in PBS as detailed above. CD-1 mice (Charles River/NCI, Frederick, MD) were injected via the intramuscular (i.m.) route with 10^5^, 10^7^, or 10^9^ CFU of pre-labeled PAO1 or PAO1TK in 50 µL into the left and right fore-limbs and hind-limbs and were immediately imaged. Imaging experiments were conducted as detailed in a previous report^19^ with some modifications. Mice were anesthetized with isoflurane (4% induction, 2% maintenance) delivered in oxygen. Each imaging session consisted of a 15 min PET scan followed by a 5 min CT scan. An equal volume of the *in vitro* labeled bacteria, 50 µL, was placed in the Gamma counter to confirm labeling of the cells used for infection.

### *In vivo* PET imaging of *P. aeruginosa* in the thigh infection model and evaluation of therapeutics

*In vivo* infection and PET imaging of PAO1 and PAO1TK were carried out in female BALB/c mice (20 g; Charles River/NCI, Frederick, MD) that were injected via the i.m. route with 10^7^ or 10^9^ CFU, as described above. After 2 h, the infected mice were injected via the i.v. route with ~250 µCi [^18^F]FIAU and were PET/CT imaged at chosen time intervals as needed for up to 5 h post radiotracer injection.

To assess the utility of PET imaging to evaluate a therapeutic against PAO1TK, infected mice were treated with ciprofloxacin prior to imaging. Briefly, mice were infected i.m. with 10^9^ CFU PAO1TK and treated with ciprofloxacin (40 mg/kg) or an equal volume of saline intraperitoneally at 5 and 75 min post-infection. At 2 h post-infection, [^18^F]FIAU was administered via i.v. injection and mice were monitored for an additional 2 h prior to PET/CT imaging. Post-imaging, mice were euthanized and the thigh sample was collected placed in 2 mL homogenizer tube (Precellys® Lysis tissue Homogenizing CKMix, Bertin Technologies) with 1 mL of PBS and homogenized using Precellys24 homogenizer (Bertin Technologies) at 5,000 rpm for 20 sec. The homogenates were serially diluted and plated on LB agar to enumerate bacteria.

## Electronic supplementary material

Supplementary information
