# Supplementary material for: Bioengineering of bacterial pathogens for noninvasive imaging and in vivo evaluation of therapeutics
Source: Sci Rep. 2018 Aug 22;8:12618. doi: 10.1038/s41598-018-30806-x (PMC6105664; doi:10.1038/s41598-018-30806-x)

Supplementary Materials

Bioengineering of bacterial pathogens for noninvasive imaging and *in vivo* evaluation of therapeutics

Authors: Sathish Rajamani[1]*, Kyle Kuszpit[1], Jennifer M. Scarff[1], Linnea Lundh[1], Maisha Khan[1], Jennifer Brown[1], Robert Stafford[1], Lisa H. Cazares[1], Rekha G. Panchal[1] and Thomas Bocan[1]*

Affiliations:
[1]Molecular & Translational Sciences Division, US Army Medical Research Institute of Infectious Diseases, 1425 Porter Street, Frederick, MD 21702

Supplementary Information Data 1. DNA sequences of Promoter – thymidine kinase fusion in *P. aeruginosa*

***P. aeruginosa* Promoter_lasR** (428bp**,** GenBank: M59425.1) **+ *E. coli* TK (**GenBank: DQ384607.1**) fusion for pUC18T-mini-Tn7T-Gm** (Genbank: AY599232.2) **cloning for *P. aeruginosa* chromosomal integration (BamHI and HindIII)**

GGATCCcgctgtgcctttgcgcgtgcgccgcgcacagacgtctgcgcctcggatcgcccggccgagagcggtcgcacgcgtggc
gatgggccgacagtgaacccggggaccaggtgtgactgggtattcagttcgcataaaatgtgatctagatcacatttaaacgtttgcttaccct
ctaggacgggtatcgtactaggtgcatcaaacgctgcggtctattgttaagtgggactgaaatgtgcctttccggcacaacgccaactctata
gagtgggctgactggacatcttcaggggtcgtcgggcacgggcgcatgcgcctcggcaggaagccgggattctcggactgccgtacaac
gtgccggatatcgggtgccgaatccatatttggctgattggttaatagtttaagaagaacgtagcgctatATCGATatggcacagctatat
ttctactattccgcaatgaatgcgggtaagtctacagcattgttgcaatcttcatacaattaccaggaacgcggcatgcgcactgtcgtatatac
ggcagaaattgatgatcgctttggtgccgggaaagtcagttcgcgtataggtttgtcatcgcctgcaaaattatttaaccaaaattcatcattattt
gatgagattcgtgcggaacatgaacagcaggcaattcattgcgtactggttgatgaatgccagtttttaaccagacaacaagtatatgaattat
cggaggttgtcgatcaactcgatatacccgtactttgttatggtttacgtaccgattttcgaggtgaattatttattggcagccaatacttactggc
atggtccgacaaactggttgaattaaaaaccatctgtttttgtggccgtaaagcaagcatggtgctgcgtcttgatcaagcaggcagaccttat
aacgaaggtgagcaggtggtaattggtggtaatgaacgatacgtttctgtatgccgtaaacactataaagaggcgttacaagtcgactcatta
acggctattcaggaaaggcatcgccacgattaa**AAGCTT**

Sequentially cloned into pBBR1MCS5 following PCR with below primers and cloning with desired restriction sites.

5'ProLasR w/KpnI
GAAACGGTACC CGCTGTGCCTTTGCGCGTG

3' ProLasR w/ClaI
GAAACATCGATAGCGCTACGTTCTTCTTAAACTATTAACCAATCAGCC

5'E.coli_TK w/ClaI
CAAACATCGATATGGCACAGCTATATTTCTACTATTCCGCAATGAATGC

3'E.coli_TK w/XbaI
CAAACTCTAGATTAATCGTGGCGATGCCTTTCCTGAATAGC

Primers for PCR amplification and subcloning of PromoterLasR-TK into pUC18T-mini-Tn7T-Gm vector using pBBR1MCS5-PlasR-TK plasmid template

5'ProLasR w/BamHI
GAAACGGATCCCGCTGTGCCTTTGCGCGTG

3'E.coli_TK w/HindIII
CAAACAAGCTTTTAATCGTGGCGATGCCTTTCCTGAATAGC

Supplementary Information Data 2. DNA sequences of Promoter – thymidine kinase fusion in *A. baumannii*

***A. baumannii* Promoter_16srDNA (A1S_r01) promoter** (327bp, GenBank: CP000521.1) +
pantoate--beta-alanine ligase (vitB5 pathway) Ribosome Binding Site and *E. coli* TK

GGATCCgattagattggttgctttaagtgatgaatttgatgatgagatggtttagagactaagttttaaaaatatcgaaattacctgttgacttt
taagagattaagagtaatatagccgacctagcttgctggtgacgaaccagtaagaagatcattaagagaattgaagaacaacttgtgtggatt
tttactgattgattaatcgaaataattttcattgattgattggtttaaattactcgaagtttatttgagcgaaatttaagtcagtaattgatgagccaga
attggcaccttgtctataaataaggtgcaaaatgattttaactgacacactttccaagttgagctgtaattcATCGATatggcacagctatatt
tctactattccgcaatgaatgcgggtaagtctacagcattgttgcaatcttcatacaattaccaggaacgcggcatgcgcactgtcgtatatacg
gcagaaattgatgatcgctttggtgccgggaaagtcagttcgcgtataggtttgtcatcgcctgcaaaattatttaaccaaaattcatcattatttg
atgagattcgtgcggaacatgaacagcaggcaattcattgcgtactggttgatgaatgccagtttttaaccagacaacaagtatatgaattatc
ggaggttgtcgatcaactcgatatacccgtactttgttatggtttacgtaccgattttcgaggtgaattatttattggcagccaatacttactggcat
ggtccgacaaactggttgaattaaaaaccatctgtttttgtggccgtaaagcaagcatggtgctgcgtcttgatcaagcaggcagaccttataa
cgaaggtgagcaggtggtaattggtggtaatgaacgatacgtttctgtatgccgtaaacactataaagaggcgttacaagtcgactcattaac
ggctattcaggaaaggcatcgccacgattaaAAGCTT

Primers for Prrn_RBS-TK cloning into pUC18T-mini-Tn7T-Gm vector using pUC57-Prrn_RBS-TK

5’Pro_16S_AB w/BamHI
GAAACGGATCCGATTAGATTGGTTGCTTTAAGTGATGAATTTGATGATG

3'E.coli_TK w/HindIII
CAAACAAGCTTTTAATCGTGGCGATGCCTTTCCTGAATAGC

PTn7R (Chromosomal integration verification)
GTAAACTGAAATCAGTCCAGTTATGCTGTGA

PglmSF1 (Chromosomal integration verification)
TTCGTTTTTGCTGATGAAAATAGCGGTG

Supplementary Information Data 3. DNA sequences of Promoter – thymidine kinase fusion in Bp82

***B. pseudomallei* S12 gene promoter (**205bp, GenBank: CP004379.1**) + *B. pseudomallei* codon-optimized *E. coli* TK gene fusions for gene synthesis and cloning into pUC18TminiTn7-Km::FRT (**GenBank: EU223384.2**)**

BpS12 promoter fusion and with protein secretory signal sequences for bacterial TK secretion into host cell. Codon optimized of *E. coli* TK for Bp engineering was carried out using http://www.jcat.de/

GGATCCcgcctttgcattccggcaccaaaaatcacgcaagctgttgactcgcttgggattttcggaatatcatgccaggttccggttctcgg
tgggttgattcctgggcgcaagctcaggcgtcgcgcacgcgtttcggagcgtcacgcacgtcggatttcgctttgcccgagttcgacgtgtc
gattttggtcaatttcaggaataaacaACCGGTATGGCGCAGCTGTACTTCTACTACTCGGCGATGAACGCGGGCA
AGTCGACGGCGCTGCTGCAGTCGTCGTACAACTACCAGGAGCGCGGCATGCGCACGGTGGTGTACACG
GCGGAGATCGACGACCGCTTCGGCGCGGGCAAGGTGTCGTCGCGCATCGGCCTGTCGTCGCCGGCGAA
GCTGTTCAACCAGAACTCGTCGCTGTTCGACGAGATCCGCGCGGAGCACGAGCAGCAGGCGATCCACT
GCGTGCTGGTGGACGAGTGCCAGTTCCTGACGCGCCAGCAGGTGTACGAGCTGTCGGAGGTGGTGGAC
CAGCTGGACATCCCGGTGCTGTGCTACGGCCTGCGCACGGACTTCCGCGGCGAGCTGTTCATCGGCTC
GCAGTACCTGCTGGCGTGGTCGGACAAGCTGGTGGAGCTGAAGACGATCTGCTTCTGCGGCCGCAAGG
CGTCGATGGTGCTGCGCCTGGACCAGGCGGGCCGCCCGTACAACGAGGGCGAGCAGGTGGTGATCGG
CGGCAACGAGCGCTACGTGTCGGTGTGCCGCAAGCACTACAAGGAGGCGCTGCAGGTGGACTCGCTG
ACGGCGATCCAGGAGCGCCACCGCCACGACTAAAAGCTT

Primers for PCR amplification of ProS12-TK promoter fusions into pUC18TminiTn7-Km::FRT using pUC57 clones (Genewiz DNA synthesis) of above inserts

5'NewBpProS12 w/KpnI
CAAATGGTACCGGA TCC CGC CTT TGC ATT CCG G

3'TKBPcodw/HindIII
CAAATAAGCTTTTAGTCGTGGCGGTGGCG

Supplementary Information Figure 1. Growth inhibition studies with WT and *tk* engineered *Acinetobacter baumannii* ATCC 17978 in the presence of Zidovudine and FIAU

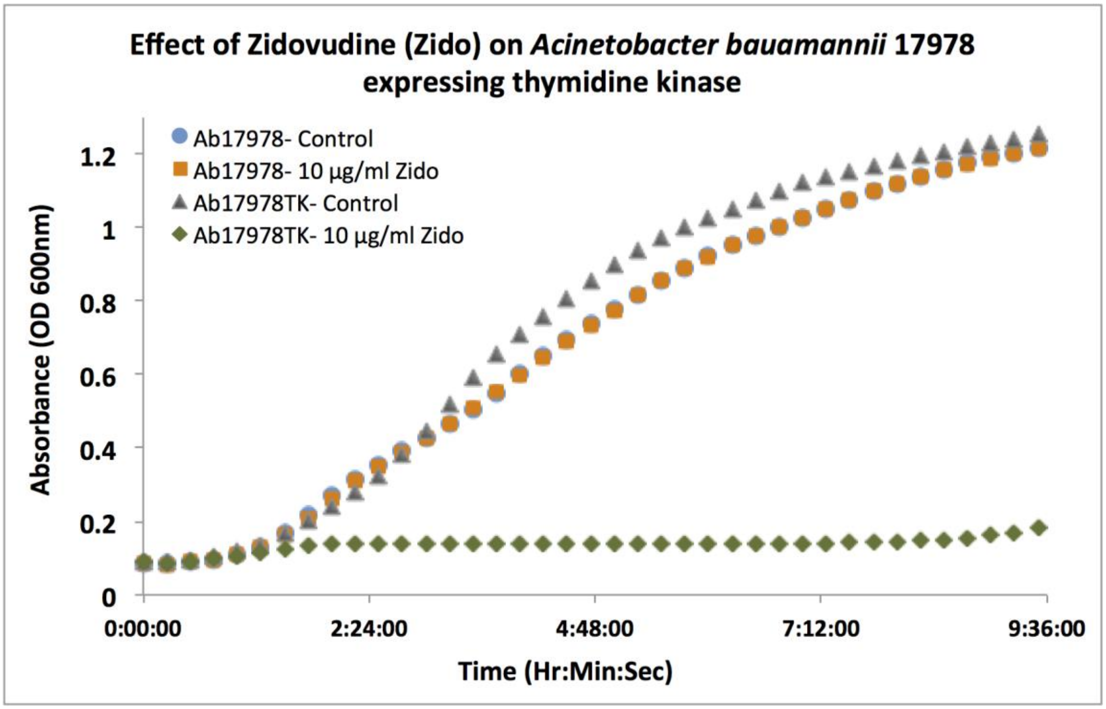

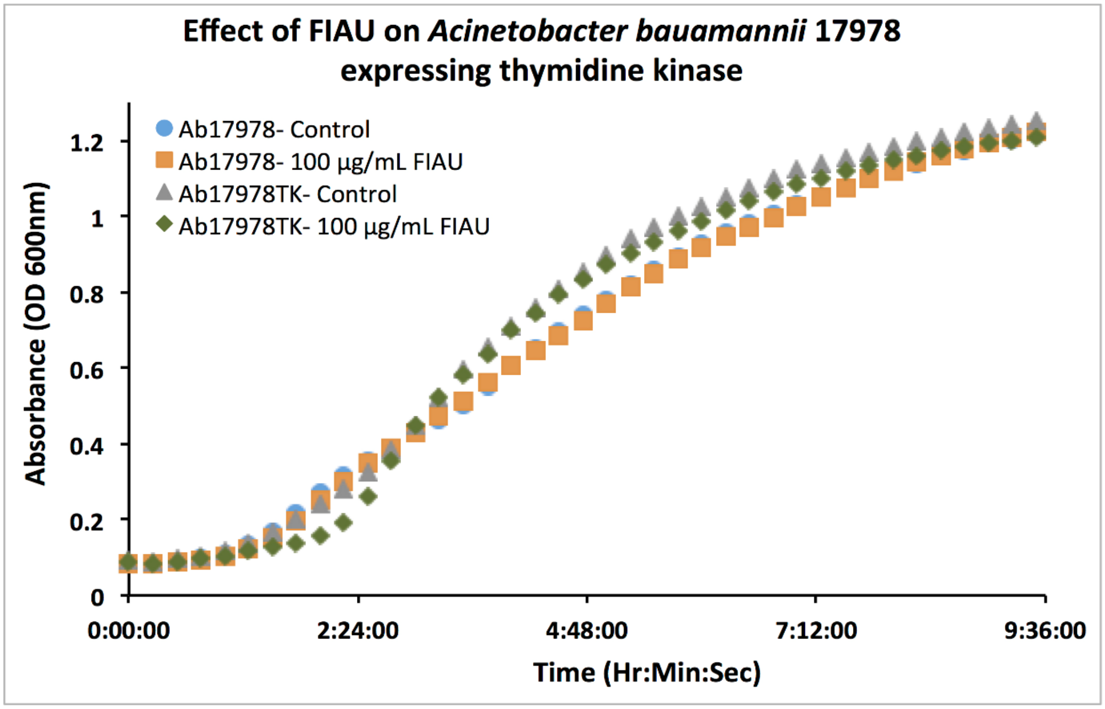

Supplementary Information Figure 2. Growth inhibition studies with WT and *tk* engineered *B. pseudomallei* Bp82 in the presence of Zidovudine and FIAU

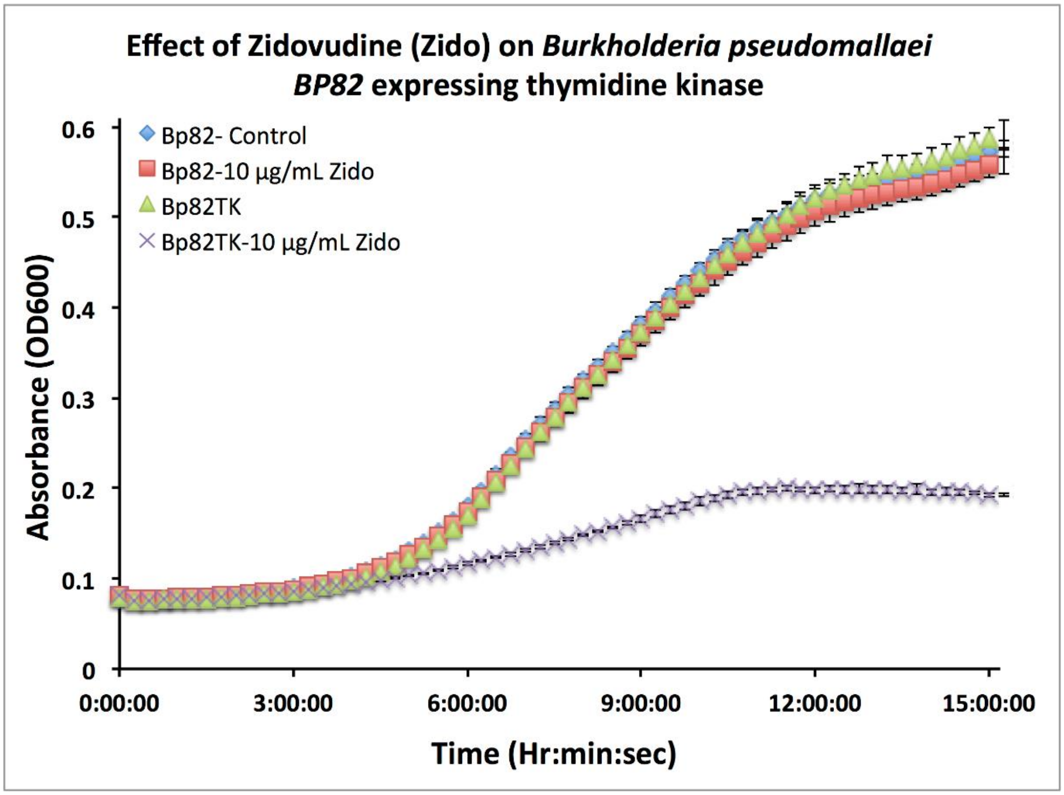

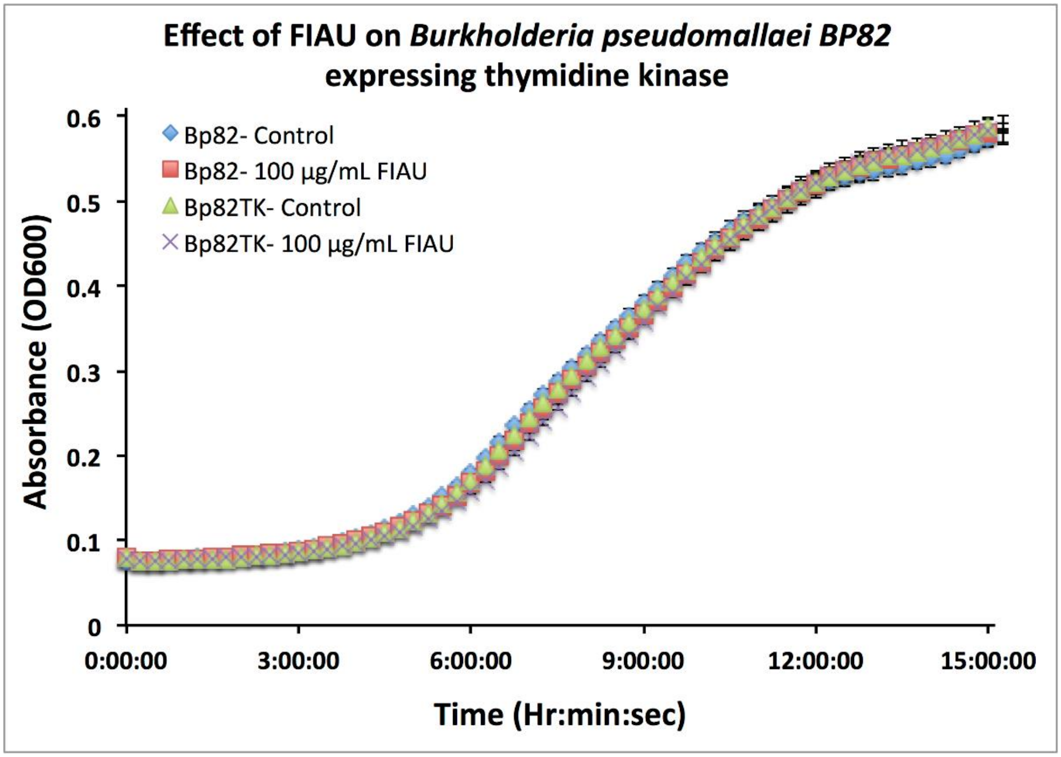

Supplementary Information Figure 3. Dynamic PET/CT scans with PAO1 and PAO1TK infected mice

In vivo images of mice (m03, upper panel, and m04, lower panel) injected with 10^9 CFU PAO1TK (left upper and lower quadrants) and PAO1 (right upper and lower quadrants) and imaged at different time points after [^{18}F]FIAU injection. Mouse m03 images are threshold adjusted to focus on the upper limbs injected with bacteria, while m04 is threshold adjusted to show lower limbs with bacteria. Table below shows the volume-of-interest (VOI) and signal intensities (UL-upper right, LL-lower right UR-upper right, LR- lower right, and BG-background). The signal intensities were used to tabulate the corresponding signal-to-noise ratios**.**

To quantify the PET data, a sphere equivalent to a 50 µl volume was used so that the sample size was the same for each treatment. The 50 µl sphere or VOI was applied to the images and the amount of radioactivity quantified. The background signal was defined as the amount of PET signal present in the VOI applied to the muscle in the non-infected limb, e.g., in mouse 03, background (BG) equals 4.61 %ID/g at 1 h10min. For the determination of the bacterial-tk signal, the 50 µl VOI was applied to the limb containing the bacteria expressing thymidine kinase, e.g., mouse 03 signal in the upper right limb (UL) was 6.06 %ID/gm. The signal-to-noise was the ratio of the activity in the UL divided by BG or 6.06/4.61 which equals 1.32.

In vivo images of mouse (m03) with 10^9 PAO1 (WT) and PA01TK (TK)

~1h10m ~2h30m ~3h50m ~4h55m

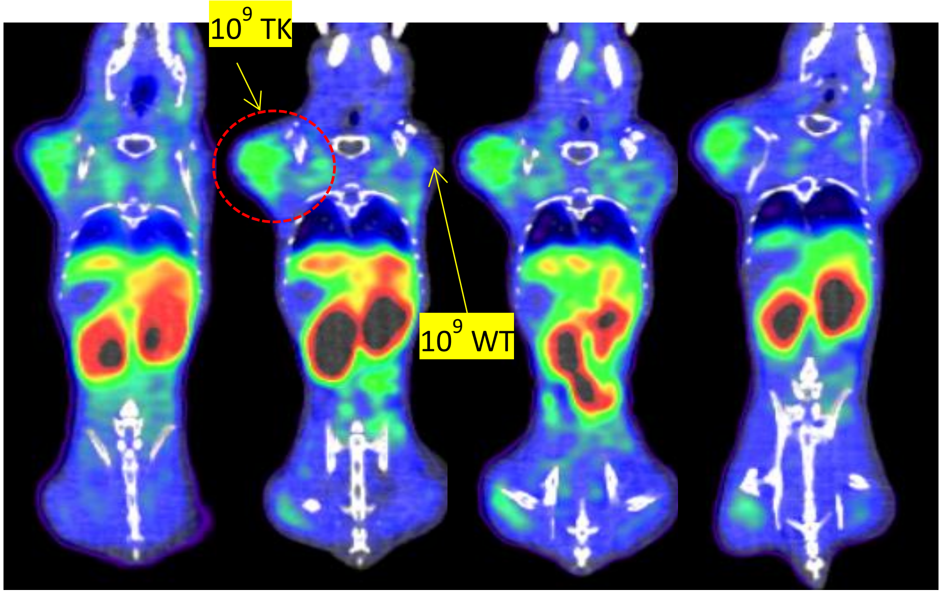

~50µL VOIs used for analysis

m03	Mean (nCi/cc)			Sig to Noise	
Scan @	UL	LL	BG	UL-BG	LL-BG
~1h10m	12219.90	11734.20	9287.46	1.32	1.26
~2h30m	7558.64	7490.90	5017.08	1.51	1.49
~3h50m	4569.47	4656.05	2934.45	1.56	1.59
~4h55m	3157.26	2922.62	2134.91	1.48	1.37

	Decay Corrected				
m03	Mean (%ID/g)			Sig to Noise	
Scan @	UL	LL	BG	UL-BG	LL-BG
~1h10m	6.06	5.82	4.61	1.32	1.26
~2h30m	6.23	6.18	4.14	1.51	1.49
~3h50m	6.36	6.48	4.08	1.56	1.59
~4h55m	6.49	6.01	4.39	1.48	1.37

In vivo images of mouse (m04) with 10^9 PAO1 (WT) and PA01TK (TK)

~1h10m ~2h30m ~3h50m ~4h55m

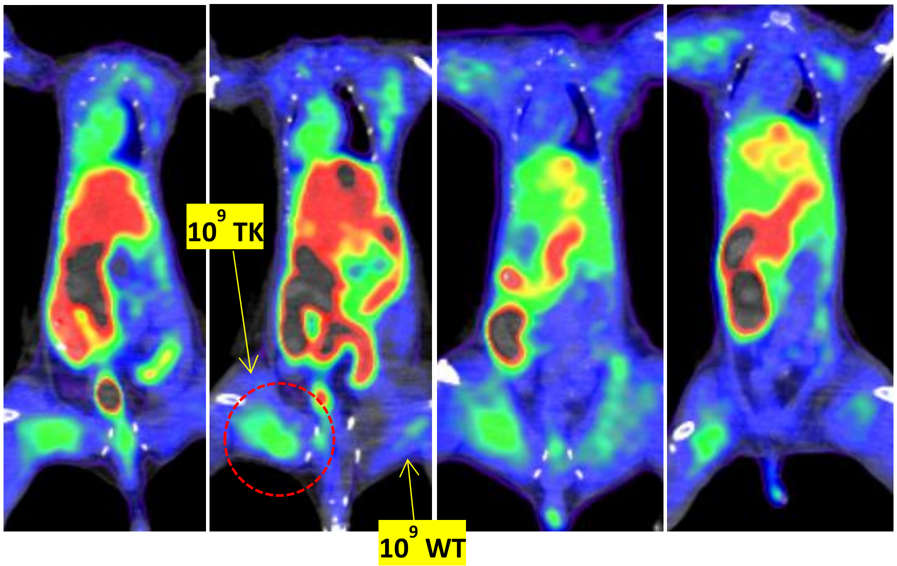

~50µL VOIs used for analysis

m04	Mean (nCi/cc)			Sig to Noise	
Scan @	UL	LL	BG	UL-BG	LL-BG
~1h10m	11556.00	11203.40	7802.61	1.48	1.44
~2h30m	7567.50	7022.72	3951.73	1.91	1.78
~3h50m	4683.13	4512.75	2399.43	1.95	1.88
~4h55m	3064.61	3006.66	2024.68	1.51	1.49

	Decay Corrected				
m04	Mean (%ID/g)			Sig to Noise	
Scan @	UL	LL	BG	UL-BG	LL-BG
~1h10m	5.72	5.55	3.86	1.48	1.44
~2h30m	6.23	5.78	3.25	1.91	1.78
~3h50m	6.50	6.26	3.33	1.95	1.88
~4h55m	6.29	6.17	4.15	1.51	1.49

Note: Animals m03 and m04 both died just prior to scanning at ~4h55m

Supplementary Information Figure 4: Bacterial imaging in BALB/c after infection with 10^7 CFU PAO1 (WT) or PAO1TK (TK) detected with [^{18}F]FIAU

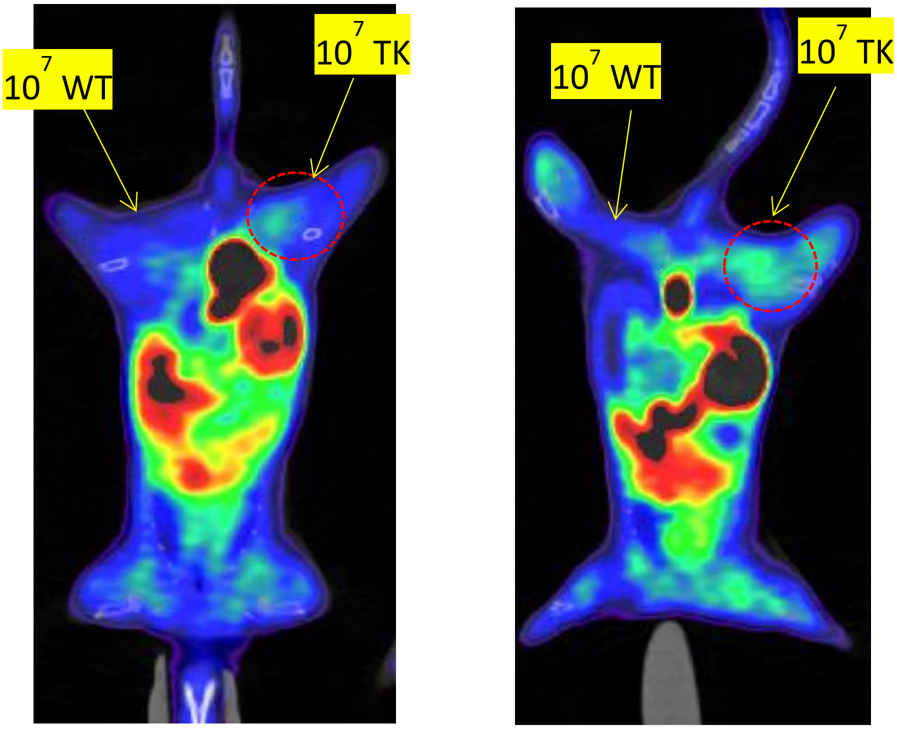

Supplement: Supplementary file 1 — Supplementary information [file 41598_2018_30806_MOESM1_ESM.pdf]
